# Mediating Effect of Hope on the Relationship Between Depression and Recovery in Persons With Schizophrenia

**DOI:** 10.3389/fpsyt.2021.627588

**Published:** 2021-02-09

**Authors:** Sri Padma Sari, Murti Agustin, Diyan Yuli Wijayanti, Widodo Sarjana, Umi Afrikhah, Kwisoon Choe

**Affiliations:** ^1^Department of Nursing, Faculty of Medicine, Universitas Diponegoro, Semarang, Indonesia; ^2^Department of Nursing, Chung-Ang University, Seoul, South Korea

**Keywords:** depression, hope, moderating effect, recovery, schizophrenia depression, mediating effect

## Abstract

**Background:** Depression and hope are considered pivotal variables in the recovery process of people with schizophrenia.

**Aim:** This study examined the moderating effect of depression on the relationship between hope and recovery, and the mediating effect of hope on the relationship between depression and recovery in persons with schizophrenia.

**Methods:** The model was tested empirically using the data of 115 persons with schizophrenia from Central Java Province, Indonesia. The Calgary Depression Scale for Schizophrenia, Schizophrenia Hope Scale-9, and Recovery Assessment Scale were used to measure participants' depression, hope, and recovery, respectively.

**Results:** The findings supported the hypothesis that depression moderates the relationship between hope and recovery, and hope mediates the relationship between depression and recovery.

**Conclusions:** The findings suggest that mental health professionals need to focus on instilling hope and reducing depression to help improve the recovery of persons with schizophrenia. Furthermore, mental health professionals should actively develop and implement programs to instill hope and continuously evaluate the effectiveness of the interventions, particularly in community-based and in-patient mental health settings.

## Introduction

Globally, schizophrenia is a serious mental illness that places a prolonged burden on people of all ages and both sexes (1). The diagnosis of mental illnesses, such as schizophrenia, in Indonesia rose to 7.0% per million in 2018 from 1.7% per million in 2013 (2).

The prominent symptoms of schizophrenia include hallucinations, delusions, social withdrawal, and lack of motivation (3); however, persons with schizophrenia are also likely to experience depression (4–6). Previous studies on schizophrenia or schizoaffective disorder found that 31–59% of participants experienced depression (7–9).

Depression is common in persons with schizophrenia; however, it is underdiagnosed and undertreated (5, 9, 10). Of concern, depression in persons with schizophrenia is a significant predictor of suicidal ideation (11) and suicide (5, 9, 12). Additionally, depression lowers the quality of life of persons with schizophrenia (13, 14). Thus, depression could be a barrier to the recovery of persons with schizophrenia, whereas hope could be used to support their recovery process (15).

Recovery is a process in which an individual with mental illness finds meaning and builds a life beyond their illness (16). Hope plays an important role in the recovery of persons with mental illnesses (17, 18). Previous studies found that a high level of hope was associated with a high quality of life in persons with schizophrenia (14, 19). Moreover, depression indirectly mediated the relationship between hope and quality of life (20).

According to the illness identity model (21, 22), a stigmatized view of mental illness diminishes hope and self-esteem, which induces detrimental effects, such as depressed mood and suicidal ideation, and eventually hinders recovery among people with severe mental illnesses. The model suggests that hope and depression are crucial variables in the recovery process of persons with schizophrenia. To the best of our knowledge, little is known about the moderating effect of depression and the mediating effect of hope on recovery. Therefore, this study aimed to examine the moderating effect of depression on the relationship between hope and recovery, and the mediating effect of hope on the relationship between depression and recovery in persons with schizophrenia in Indonesia.

## Materials and Methods

### Study Design and Participants

This study used a cross-sectional design. Persons with schizophrenia were recruited through convenience sampling from psychiatric hospitals in Central Java Province, Indonesia. Inclusion criteria were as follows: (1) aged 18–60; (2) diagnosis of schizophrenia according to the 10th revision of the International Classification of Diseases (ICD-10) and Diagnostic and Statistical Manual of Mental Disorders, Fifth Edition (DSM-5) (3); (3) psychological stability to answer the questionnaires (as determined by the medical staff); and (4) being able to provide informed consent voluntarily. The exclusion criteria were as follows: (1) intellectual disability or organic mental disorder and (2) worsening of psychotic symptoms during the data collection period. All participants in this study were assured of confidentiality and anonymity.

### Instruments

#### Socio-Demographic Questionnaire

The socio-demographic questionnaire included items on age, sex, marital status, education, occupational status, religion, and diagnosis of schizophrenia.

#### Depression

Depression was measured using the Calgary Depression Scale for Schizophrenia (CDSS) (23). The CDSS is an observer-rated interview-based instrument comprising nine items: depression, hopelessness, self-depreciation, guilty ideas of reference, pathological guilt, morning depression, early wakening, suicide, and observed depression. Each item is rated on a four-point scale (0 = absent, 1 = mild, 2 = moderate, 3 = severe) with a score range of 0–27. The CDSS is the most reliable and valid measurement of depressive symptoms in persons with schizophrenia (24). For this scale, Cronbach's alpha was 0.81.

#### Hope

Hope was measured using the Schizophrenia Hope Scale-9 (SHS-9) (25). The SHS-9 measures hope in persons with schizophrenia based on three dimensions: positive expectations for the future, confidence in life and the future, and meaning in life. This self-reported scale consists of nine items on a three-point scale (0 = disagree, 1 = agree, 2 = strongly agree). Participants can score between 0–18, with higher scores indicating higher levels of hope. For this scale, Cronbach's alpha was 0.91.

#### Recovery

The 24-item Recovery Assessment Scale-revised (RAS-R), derived from the original 41-item scale (26), is a self-administered questionnaire that includes five factors: personal confidence and hope, willingness to ask for help, goals and success orientation, reliance on others, and no domination by symptoms. This questionnaire uses a five-point scale (1 = strongly disagree; 5 = strongly agree) to evaluate the recovery status of persons with a mental illness, such as schizophrenia (score range = 24–120). For this scale, Cronbach's alpha was 0.90.

### Data Collection

This study was approved by the institutional review board of the Faculty of Medicine, Diponegoro University (No. 351/EC/FK-RSDK/V/2018). Data collection was conducted from April to June 2018 by the authors who visited the psychiatric hospitals to administer the questionnaire and the scales. Written informed consent was obtained from each participant. The questionnaire took 10 min on average to complete. After 28 participants were removed from the data due to incomplete questionnaires, the data of 115 participants were statistically analyzed.

### Statistical Analysis

The data were analyzed using SPSS Statistics 25.0 (IBM Corp., Armonk, NY). The socio-demographic characteristics of the participants were reported in terms of frequencies, percentages, means, and standard deviations (*SD*). The Kolmogorov–Smirnov test was used to examine the normal distribution of the quantitative variables; the total scores on the CDSS and SHS-9 were not normally distributed (*p* < 0.05). Therefore, we used the Mann–Whitney *U*-test to compare the groups. The scores of the RAS-R showed a normal distribution for sex and marital status; therefore, a *t*-test was used to analyze the difference between the two groups. Spearman's correlation coefficient was used to evaluate the association between the scores. Subsequently, a multiple linear regression analysis was conducted to test whether depression moderated the relationship between hope and recovery. Interaction terms hope (SHS-9) × depression (CDSS) were created and entered after adjusting for control variables. The statistical significance level was set at 0.05.

## Results

### General Characteristics and Univariate Analyses

Of the 115 participants, 73 (63.5%) were men and 42 (36.5%) were women. The mean age was 33.17 years (range: 18–70 years). Of the total participants, 30.5% had an educational level lower than elementary school, 94.8% identified as Muslim, 55.7% were single, and 64.3% were unemployed (Table 1). The mean scores of hope, recovery, and depression were 9.85 (*SD* = 3.62), 77.20 (*SD* = 5.69), and 9.31 (*SD* = 6.38), respectively (Table 1). There was no significant difference in hope, recovery, and depression according to participants' sex, marital status, and occupational status (Table 2).

**Table 1:** General characteristics of the participants (*N* = 115).

	***N* (%)**	**Mean (standard deviation)**
Sex		
Women	42 (36.5%)	
Men	73 (63.5%)	
Age (years)		33.17 (10.43)
Education		
No schooling	4 (3.5%)	
Elementary school	31 (27.0%)	
Junior high school	28 (24.3%)	
Senior high school	41 (35.7%)	
Bachelor's degree	11 (9.6%)	
Religion		
Catholic	2 (1.7%)	
Protestant	4 (3.5%)	
Muslim	109 (94.8%)	
Marital Status		
Single	64 (55.7%)	
Married	51 (44.3%)	
Occupational Status		
Unemployed	74 (64.3%)	
Employed	41 (35.7%)	
Hope		9.85 (3.62)
Recovery		77.20 (5.69)
Depression		9.31 (6.38)

**Table 2:** Univariate analyses between demographic characteristics, hope, recovery, and depression (*N* = 115).

	***N***	**Hope (Mean rank)**	***U***	***Z***	***P***	**Recovery (Mean rank or Mean)**	***U* (or *t*)**	***Z***	***P***	**Depression (Mean rank)**	***U***	***Z***	***P***
Sex			1,428.000	−0.621	0.535		−0.014		0.989		1,361.500	−0.999	0.318
Women	42	55.50				77.19				62.08			
Men	73	59.44				77.21				55.65			
Marital Status			1,619.500	−0.072	0.943		0.566		0.573		1,496.500	−0.765	0.444
Single	64	58.20				77.47				55.88			
Married	51	57.75				76.86				60.66			
Occupational Status			1,506.000	−0.065	0.948		1,511.500	−0.032	0.974		1,472.000	−0.264	0.792
Unemployed	74	57.85				57.93				57.39			
Employed	41	58.27				58.13				59.10			

### Correlation Analysis

A strong positive correlation was found between hope and recovery (*r* = 0.641, *p* < 0.001), while negative correlations were found between hope and depression (*r* = −0.440, *p* < 0.001) and recovery and depression (*r* = −0.368, *p* < 0.001) (Table 3).

**Table 3:** Spearman's correlation between hope, recovery, and depression (*N* = 115).

	**1**	**2**	**3**
1. Hope	1		
2. Recovery	0.641^**^	1	
3. Depression	−0.440^**^	−0.368^**^	1

** *p < 0.01*.

### Multiple Linear Regression Analysis

As hypothesized, depression moderated the relationship between hope and recovery. The interaction effect of depression on the relationship between hope and recovery was statistically significant (β = 0.153, *p* = 0.045). The power was 53.7% in model 4 (Table 4). Therefore, depression had a moderating effect on the relationship between hope and recovery.

**Table 4:** Moderating effect of depression on the relationship between hope and recovery (*N* = 115).

	**Step 1**		**Step 2**		**Step 3**		**Step 4**	
	**β**	***P*-value**	**β**	***P*-value**	**β**	***P*-value**	**β**	***P*-value**
Sex	−0.017	0.867	−0.025	0.727	−0.027	0.706	−0.034	0.635
Marital status	−0.058	0.558	−0.034	0.625	−0.023	0.746	−0.041	0.560
Occupational status	−0.111	0.912	−0.002	0.983	−0.007	0.915	−0.007	0.921
Hope			0.712	<0.001	0.672	<0.001	0.748	<0.001
Depression					−0.111	0.126	−0.063	0.402
Hope × Depression							0.153	0.045
*F*	0.116	0.951	28.507	<0.001	23.566	<0.001	20.889	<0.001
*R*^2^	0.003		0.509		0.519		0.537	
Adjusted *R*^2^	−0.024		0.491		0.497		0.511	
Δ*R*^2^	0.003	0.951	0.506	<0.001	0.010	0.126	0.018	0.045

As shown in Table 5, hope mediated the relationship between depression and recovery. On the second step, depression significantly predicted hope (β = −0.369, *p* < 0.001). On the third step, depression also significantly predicted recovery (β = −0.359, *p* < 0.001). On the fourth step, depression and hope were added; depression did not predict recovery (β = −0.111, *p* = 0.126), but hope significantly predicted recovery (β = 0.672, *p* < 0.001). Therefore, hope had a mediating effect on the relationship between depression and recovery. The statistical significance of the mediation effect was assessed using the Sobel test (*z* = −3.78, *p* < 0.001).

**Table 5:** Mediating effect of hope on the relationship between depression and recovery (*N* = 115).

	**Step 1**		**Step 2**		**Step 3**		**Step 4**	
	**β**	***P*-value**	**β**	***P*-value**	**β**	***P*-value**	**β**	***P*-value**
Sex	−0.017	0.867	0.004	0.970	−0.025	0.798	−0.027	0.706
Marital status	−0.058	0.558	0.009	0.922	−0.017	0.860	−0.023	0.746
Occupational status	−0.011	0.912	−0.031	0.738	−0.028	0.761	−0.007	0.915
Depression			−0.369	<0.001	−0.359	<0.001	−0.111	0.126
Hope							0.672	<0.001
*F*	0.116	0.951	4.299	0.003	4.087	0.004	23.566	<0.001
*R*^2^	0.003		0.135		0.129		0.519	
Adjusted *R*^2^	−0.024		0.104		0.098		0.497	
df	3,111		4,110		4,110		5,109	

## Discussion

In the current study, we examined the moderating effect of depression and the mediating effect of hope on the recovery process among persons with schizophrenia in Central Java Province, Indonesia. To the best of our knowledge, this study is the first to examine the moderating effect of depression and the mediating effect of hope on the recovery of persons with schizophrenia. In this study, depression moderated the relationship between hope and recovery, and hope mediated the relationship between depression and recovery. The findings of this study reaffirm the influential roles of hope and depression in the recovery of persons with schizophrenia.

Hope and optimism about the future and meaning in life, including quality of life, are central themes of the recovery process in mental health (27). Not surprisingly, persons with mental illnesses who have a high level of hope have a high quality of life (14, 19). Sustaining hope by renewing it is an important process in recovery (16). Conversely, a lack of hope is associated with a low quality of life in persons with serious mental illnesses (28). Furthermore, hopelessness significantly mediates the relationship between symptoms of depression and suicidal ideation (11).

Depression is a significant predictor of psychological well-being, such as purpose in life, in persons with schizophrenia (29). Expectedly, in this study, depression affected recovery, and it was negatively correlated with hope and recovery: higher the levels of recovery in relation to hope and personal confidence, fewer the psychiatric symptoms (30). Therefore, researchers need to explore positive factors that contribute to hope and reduce depression. Mental health professionals working in the community need to increase their efforts to enhance and maintain hope as well as reduce depression in persons with schizophrenia.

The results showed that there was no significant difference in hope, depression, and recovery according to sex, marital status, and occupational status. It is generally reported that women have more depressive symptoms than men (31). Our findings are similar to those of previous studies that show that more participants with schizophrenia were men than women, unemployed than employed, and Muslims than members of other religions (8, 32). However, the effect of the general characteristics of participants with schizophrenia on recovery has not been studied sufficiently. Further studies are needed to explore how the general characteristics of persons with schizophrenia, such as sex, educational background, marital status, and occupational status, affect their hope and recovery in Indonesia.

This study had some limitations. We collected data from patients with schizophrenia admitted to some psychiatric hospitals in Central Java Province, Indonesia. Therefore, one needs to be careful while generalizing these findings for other populations. When designing this study, we did not adequately consider other contextual factors (e.g., the length of hospital stay, duration of illness, type of occupation, family presence) that could affect participants' depression, hope, and recovery.

### Clinical Implications

This study demonstrates that maintaining and enhancing hope while paying attention to depression is essential for the recovery of persons with schizophrenia. For them, recovery is about having a sense of identity to grow beyond their illness while discovering their strength and ability to pursue personal goals (33). Most of all, hope is a pivotal factor in enhancing recovery in persons with schizophrenia. Therefore, mental health professionals should actively develop and implement psychotherapies to instill hope and reduce depression, continuously evaluate the effectiveness of such interventions, and improve services, particularly in community-based and inpatient mental health settings.

## Data Availability Statement

The raw data supporting the conclusions of this article will be made available by the authors, without undue reservation.

## Ethics Statement

The studies involving human participants were reviewed and approved by the Institutional Review Board of the Faculty of Medicine, Diponegoro University (No. 351/EC/FK-RSDK/V/2018). The patients/participants provided their written informed consent to participate in this study.

## Author Contributions

SS, MA, and KC contributed to the conception and design of the study. DW, WS, and UA organized the database. KC performed the statistical analyses. SS and KC wrote the first draft of the manuscript. All authors revised, read, and approved the submitted version of the manuscript.

## Conflict of Interest

The authors declare that the research was conducted in the absence of any commercial or financial relationships that could be construed as a potential conflict of interest.

## Abbreviations

CDSS: Calgary Depression Scale for Schizophrenia
DSM-5: Diagnostic and Statistical Manual of Mental Disorders, Fifth Edition
ICD-10, International Classification of Diseases: Tenth Revision
SHS-9: Schizophrenia Hope Scale-9
RAS-R: Recovery Assessment Scale-Revised.
